# *Frontiers in Zoology *speeds up

**DOI:** 10.1186/1742-9994-2-6

**Published:** 2005-04-18

**Authors:** Jürgen Heinze, Diethard Tautz

**Affiliations:** 1 Biologie I, Universität Regensburg, D-93040 Regensburg, Germany; 2 Institute for Genetics, Weyertal 121, D-50931 Cologne, Germany

## Introduction

Only a few months after its official launch by BioMed Central, *Frontiers in Zoology *has become a widely-read journal attracting submissions of reviews and original papers on research from all fields of zoology. To ensure that peer-reviewed research is Open Access, i.e. universally and freely available online to everyone and that it is archived in internationally recognised free repositories [1], from April 1, 2005, authors of articles accepted for publication will be asked to pay an article-processing charge (APC) of £ 330.

Traditionally, readers pay to access articles, either through subscriptions or by paying a fee each time they download an article. For many journals, subscription costs have increased over the last couple of years. Journal costs have always been a problem for researchers in some countries, but the recent cost escalation together with cuts in funding for universities have almost globally resulted in libraries describing to fewer journals [2]. The availability of results to readers therefore has become more and more limited. Although traditional journals publish authors' work for free (unless there are page or colour charges), having to pay to access articles limits how many can read, use and cite them.

## The advantages of Open Access

As summarized by Slade et al. [3], the Open Access policy generally changes the way in which articles are published. First, all articles become freely and universally accessible online, and so an author's work can be read by anyone at no cost. Second, the authors hold copyright for their work and grant anyone the right to reproduce and disseminate the article, provided that it is correctly cited and no errors are introduced [1]. Third, a copy of the full text of each article is permanently archived in an online repository separate from the journal. Articles in *Frontiers in Zoology *are archived in PubMed Central [4], the US National Library of Medicine's full-text repository of life science literature, and also in repositories at the University of Potsdam [5] in Germany, at INIST [6] in France and in e-Depot [7], the National Library of the Netherlands' digital archive of all electronic publications.

Open Access has four broad benefits for science and the general public. First, authors are assured that their work is disseminated to the widest possible audience, given that there are no barriers to access their work. This is accentuated by the authors being free to reproduce and distribute their work, for example by placing it on their institution's website. It has been shown that free online articles are more highly cited because of their easier availability [8]. Second, the information available to researchers will not be limited by what their library can afford, and the widespread availability of articles will enhance literature searching [9]. Third, the results of publicly funded research will be accessible to all taxpayers and not just those with access to a library with a subscription. Note that this public accessibility may become a legal requirement in the USA if the proposed Public Access to Science Act is made law [10]. Fourth, a country's economy will not influence its scientists' ability to access articles because resource-poor countries (and institutions) will be able to read the same material as wealthier ones (although creating access to the internet is another matter [11]).

## Article-processing charges

APCs will allow continued Open Access to all articles published in *Frontiers in Zoology*. Authors are asked to pay £ 330 if their article is accepted for publication. Waiver requests will be considered on a case-by-case basis, by the Editor-in-Chief. Authors can circumvent the charge by getting their institution to become a 'member' of BioMed Central, whereby the annual membership fee covers the APCs for all authors at that institution for that year. Current members include the Deutsche Zoologische Gesellschaft, who initiated and supports this journal, Harvard, Princeton and Yale universities, all UK universities, CNRS, INRA, and Institute Pasteur, Max Planck Society, and many others [12]. Publishing in *Frontiers in Zoology *and other BMC journals is completely free of costs for all whose institutions are listed as BioMed Central Institutional Members. Please note that no charge is made for articles that are rejected after peer review. Many funding agencies have also realized the importance of Open Access publishing and have specified that their grants may be used directly to pay APCs [13].

The APC pays for efficient and thorough peer review, for the article to be freely and universally accessible in various formats online, and for the processes required for inclusion in PubMed and archiving in PubMed Central, e-Depot, Potsdam and INIST. Although some authors may consider £ 330 expensive, it must be remembered that *Frontiers in Zoology *does not levy additional page or colour charges on top of this fee, which can easily exceed £330. With the article being online only, any number of colour figures and photographs can be included, at no extra cost. Given that colour prints are often essential for the documentation of results from research on morphology, neurobiology, taxonomy etc. and extremely valuable also in other discipline of zoology, *Frontiers in Zoology *provides a suitable medium for the broad dissemination of high-quality research from all areas of zoological research.

Although several journals now offer free access to their articles online, this is different from Open Access (as defined by the Bethesda Statement [14]). Journals often delay free access for 6–12 months, and even when the full text is available, readers are not allowed to reproduce and/or disseminate the work because of restrictions imposed by the copyright policy. That said, *Frontiers in Zoology *is not alone in the move to Open Access funded by APCs. The *British Medical Journal *has recently announced that it cannot continue to provide free access to its website [14] and is considering various sources of revenue, including APCs [16]. Also, the Public Library of Science has set up new Open Access journals, and have elected to set APCs of US$1500 for each accepted article [17]. Given that the Public Library of Science has used television advertising to promote journals [10], the high profile of these journals will raise awareness of Open Access and encourage researchers in all disciplines to understand and accept Open Access, with APCs as an acceptable method to fund it.

## Conclusion

By providing a forum for Open Access, APCs will enable *Frontiers in Zoology *to continue to publish attractive and important papers on outstanding research from all fields of zoology that will be accessible to everyone worldwide. We believe this change will support the current renaissance of integrative zoology as a modern and vigorous field of research, and we hope you will support this progress by submitting your next article to this Open Access journal.
